# Combined Material Recycling Study with Aesthetic of Entropy and Place Making

**DOI:** 10.1155/2015/208342

**Published:** 2015-03-26

**Authors:** Yifeng Wen

**Affiliations:** School of Architecture and Urban Planning, Guangzhou University, Guangzhou 510006, China

## Abstract

Green building is a hot topic today. The place making and urban cultures are also important issues in postindustrial society. The industrial heritage renovation projects provide a research opportunity in combination with both aspects. This paper tries to shed new light on this issue by interdisciplinary methods, to study six Guangzhou industrial heritage renovation projects, giving aesthetic values for six sites concerning place making and culture creation, especially giving an explanation for old building material's aesthetic performance in terms of concepts “entropy” and “archetype.” The conclutions regard: the six places are brand spaces of “authentic Guangzhou” that make local experiential knowledge, emotional significance and creative communities in combination with historical and cultural narratives.

## 1. Introduction

In postindustrial society, energy efficiency, sustainable development, and recycle economics are focus issues. Creative city and urban art and culture are also hot topics in this era. But the relevant studies combined two fields to form integral approaches and theories are very few. Energy efficiency, sustainable development, and recycle economic summed up in architectural/engineering academic areas are designated as green building technologies with relevance to tool reason, while the creative city and urban culture are identified as value reason for the purpose of directly advancing people's life according to lifestyle in postindustrial era. How to conciliate those two aspects for benefit is the problem this paper tries to solve, which leads to a connection between technical views and humanities.

### 1.1. Greening Building Technology

Building sector is the main contributor to total energy/resources consumption. Tony Arnel [1], the chair of World Green Building Council, stated that buildings consumed 32% of the world's resources in construction and they accounted for 40% of global energy consumption in 2010. Many countries revealed that the building sector appeared to be the largest contributor to total energy consumption in these countries. For example, in developed countries, in the UK, the buildings accounted for nearly 40% of total energy consumption and produced 50% of all UK carbon emission [2]. In the EU, building used up an estimated 50% of the total energy consumption and contributed almost 50% CO_2_ during their life cycles [3]. Among other things, it is responsible for 40% of overall waste production in the EU [4]. In Canada, the residential and commercial buildings consumed about 30% of the total secondary energy use and contributed about 29% of CO_2_ equivalent greenhouse gas emissions [5]. Today, about 50% of the world's resources are used for buildings, and 50% of solid waste came from buildings. Energy used in building's function/operation is about 25% of the world's energy consumption, with construction related energy consumption (such as steel, cement, glass, and other building material industry energy consumption) totally being about 46.7%. Urban areas account for over 70 percent of global carbon emissions and for almost 70 percent of world energy consumption (International Energy Agency, Source: http://www.iea.org/). While unsustainable material production, human lifestyles, and consumerism lead to alterations at multiple scales in urban systems, they also generate negative effects in residents' everyday lives. All these problems demonstrate the need to reduce material and energy flows and lower environmental impacts [6–8].

The issue of monitoring the environmental impact of building processes is attracting more and more attention among civil engineers, architects, and researchers as building industry is one of the largest industry sector. In China, the concept of green building was developed from “energy-saving and land-saving residential building” required by central government in 2004. To be specific, the green building should be energy-saving, land-saving, water-saving and material-saving, environment-benign, and pollution-reducing, summarized as “Four-Saving and One-Benign.” In China, ESGB (Evaluation Standard for Green Building) sets 10% as the threshold proportion (by mass) of recyclable material (not recycled material) for all building materials. As a result, the proportions of most GBL (Green Building Label) projects concentrate in the range of 10–15% [9].

The enforcement of energy efficient building is considered as the most successful strategy for CO_2_ emission reduction and energy saving during the life cycle of the building, while the application of suitable green building products plays an important role in facilitating energy efficient building promotion. The green building product is intended to be environmentally friendly, with such characteristics as energy conservation, low emission, low toxicity, and ability to be renewable and recycled for the whole life cycle [10]. In general, the life cycle of a green building product includes the extraction of raw materials, processing of raw materials, designing and manufacturing of products, packaging and transport, installation of products into a ready building, use and maintenance, demolition and/or dissembling of the building, and disposal or recycling of the products. All this phase should be well considered to minimize the life cycle energy consumption [11]. The essence of sustainable development is to deliver social and economic development without compromising environmental quality. Material flow analysis or substance flow analysis (M/SFA) is a well-established method to assess the sustainability of socioeconomic development and environmental change, particularly from the perspective of improving material/substance flow efficiency [12].

### 1.2. Combined Material Recycling with Place Making and Urban Culture

Focusing on technological efficiency as the solution to sustainability issues in the development of the built environment has done little in the past to bridge the rifts between humanity and the natural world. If we lack understanding of the in-depth relationship between the built environment and nature, we may be misled and seduced by technology. There is the popular perception that if we assemble in one single building enough ecogadgetry such as solar collectors, photovoltaics, biological recycling systems, building automation systems, and double-skin facades, we will instantaneously have ecological architecture [13].

Some scholars recognize that the main barriers for sustainable building lie within police, process, and social aspects rather than in technology [14, 15]. A common theme has identified the major institutional and organizational barriers that hinder the construction industry's progress towards sustainability. From a survey, Hwang and Tan [16] describe it as a “vicious cycle” of hinge costs, lack of client demands, lack of research and development (R&D), and lack of collaborative efforts and communication between various stakeholders. Thus, in planning process, we must combine the technical reason with a variety of values with which various stakeholders are concerned in order to form a common sense, which directly determines the life quality for people and creates culture.

The aim of this paper is combined material recycling study with place making and creating urban culture. The rest of the paper is organised as the following five parts. First, we put forward the economic background of Guangdong and Guangzhou, to illustrate that Guangdong, in specific the Pearl River Delta region, has already entered the postindustrial society. We address the renovations of six industrial heritages in Guangzhou as case studies to demonstrate the industrial transition in this era and the situation of culture creation in these places, to usher the issues concerning material recycling, aesthetic of entropy, and place making. Second, energy efficiency and material recycling effects of the six sites are analyzed and the relevant policies are addressed. Third, beyond the traditional topic about material recycling, we explain the aesthetic of entropy and place making accounted for by old materials in terms of phenomenology, giving aesthetic performance values for the six sites. Fourth, two main limitations in this research are present in terms of facing difficulty to deal with the interdisciplinary problems across technology and humanity. Fifth, the conclusions sum up the main points of this paper and raise some new thinking for further studies.

## 2. Background and Cases Studies

**Figure 1:**
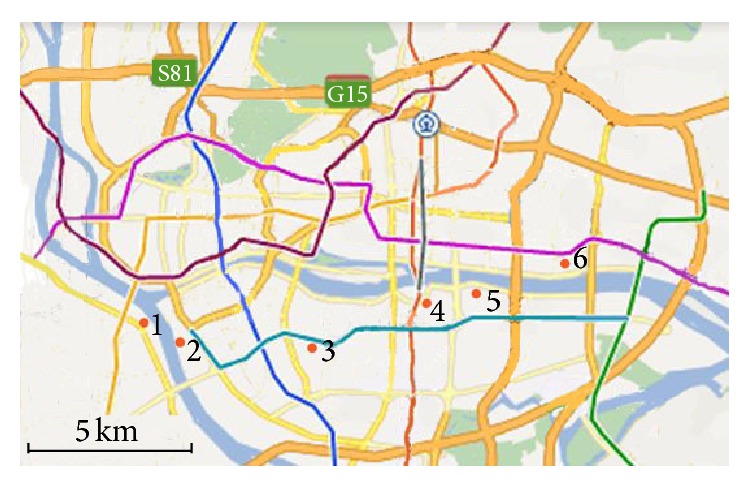
The location of six industrial heritages. Notes: (1) Xinyi Place (Xinyi International Place); (2) Taikoo Wharf; (3) Xingang 82 (Guangzhou Textile Exchange Park); (4) T.I.T Creative Park; (5) Party Pier (Zhujiang Party Pier Beer Culture & Art Zone); (6) Redtory.

In 2007, the GDP of Guangdong province has amounted to $4360, and that of the Pearl River Delta region has amounted to $7516, up to the level of middle-income countries. According to the Hollis B. Chenery's industrial structure theory and general law of developed countries, with the improvement of economic development and per capita income, service industry will usher in a speed-up development. However, in contrast, Guangdong's service industry shows a strikingly low proportion of 43.3% in 2007; while the percentages of service sector in high-income countries in the world are more than 70%, the proportions of it in middle-income countries are also more than 60%. After the 2007 financial crisis, the inherent path-dependent development of manufacturing industry in Guangdong province had a difficulty, but the government took it as an advantage to decisively adjust the industrial structure of Guangdong's economic development. Up to 2010, the percentages of tertiary industry reached 61. 01%, and in 2012 they accounted for 68.58% [17]. In recent years, Guangzhou ushered in a postindustrial period. Guangzhou's municipal government put forward a “cut down secondary industry to develop tertiary industry” policy, implementing “three old renewal projects.” The “three old” means old town, old factory building, and old villages in city. The following are six cases of industrial heritage renovation in Guangzhou for compared studies.  Figure 1 is the location of six industrial heritages.  Table 1 is the business configuration of six industrial heritages after renovation.  Table 2 is the service relationship between six industrial heritages and the statistic sectors of tertiary industries.

The scales of the six sites are defined by “400-meter rule,” which means that the interval distance between nodes of main street networks in old town is generally 400 meters. Thus, if a site's length in terms of its any side is nearly or beyond 400-meter, which means this site at least in one side fully filled and even beyond a street block, we defined it as big scale. If the distance of any side of site is smaller than 200 meters, we defined it as small. If between those two situations, we defined it as medial scale.

**Figure 2:**
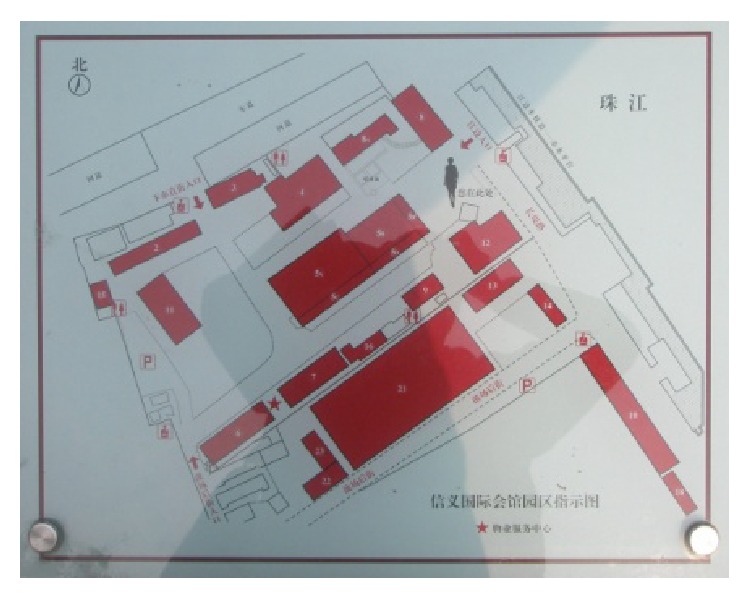
Layout of Xinyi Place.

**Figure 3:**
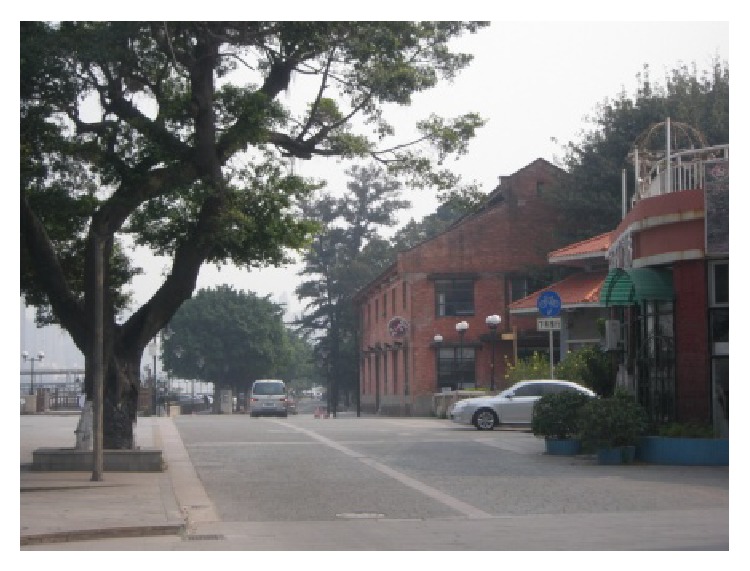
Riverside view.

**Figure 4:**
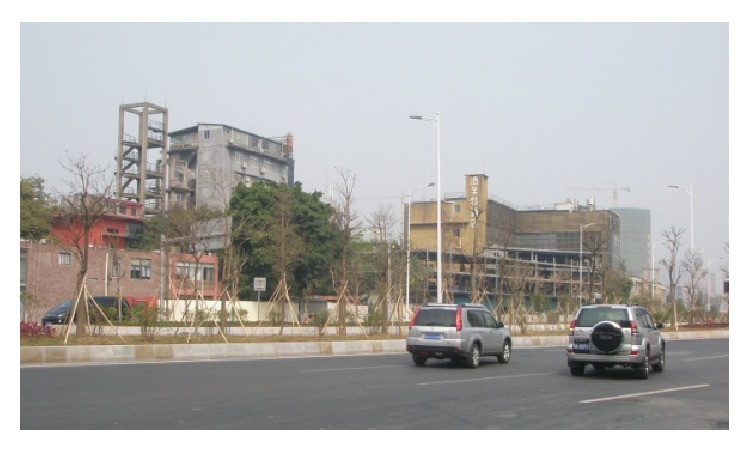
Under-construction projects.

**Figure 5:**
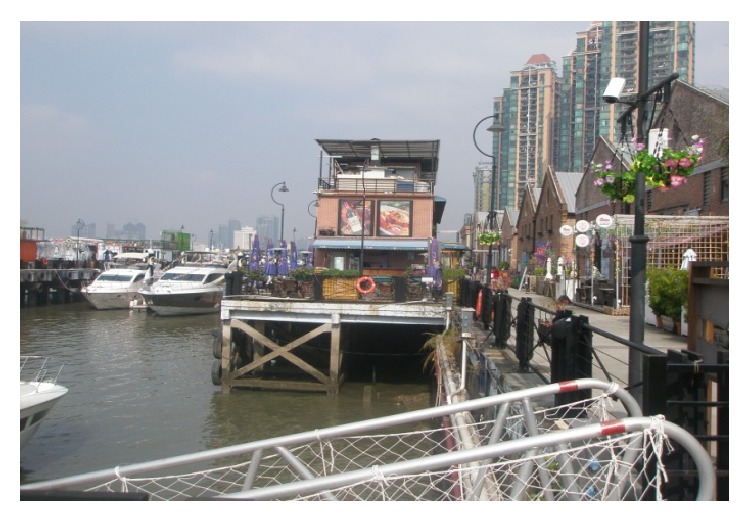
Riverside view of Taikoo Wharf.

**Figure 6:**
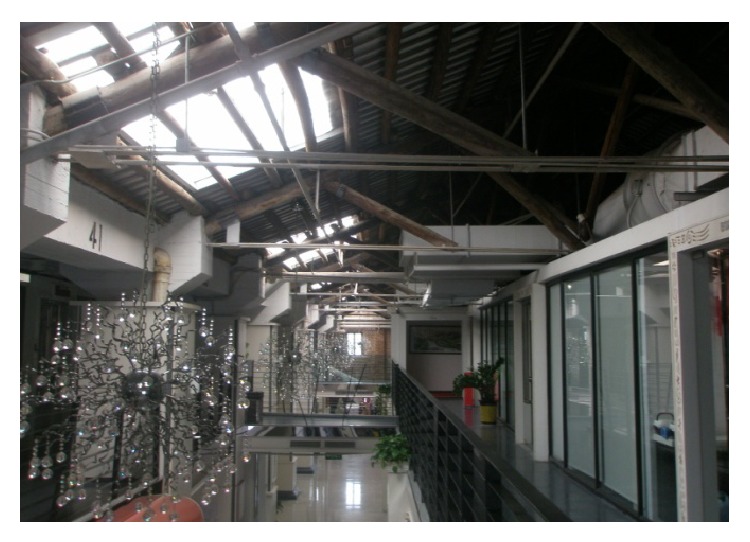
Interior decoration.

**Figure 7:**
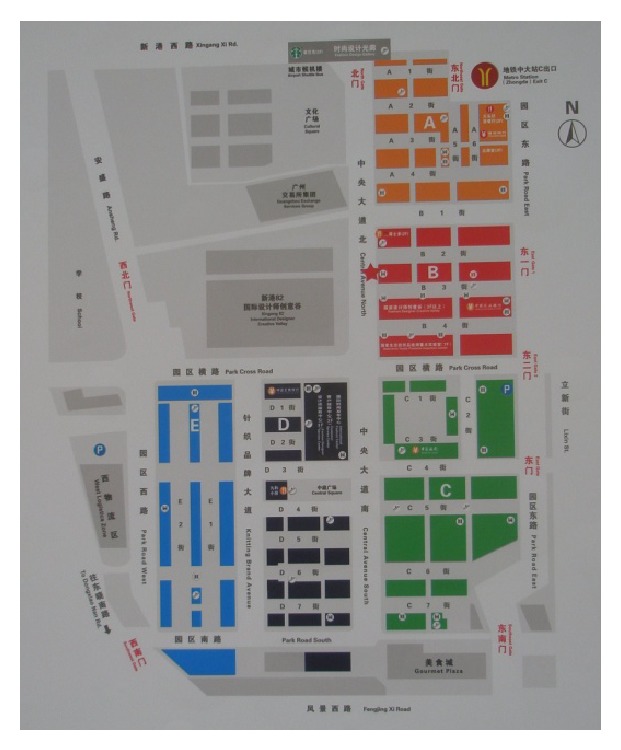
Layout of Xingang 82.

**Figure 8:**
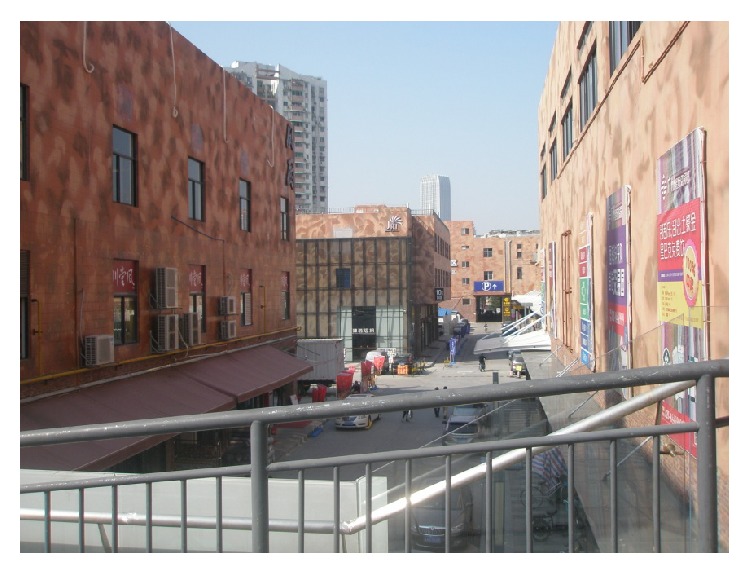
A view of Xingang 82.

**Figure 9:**
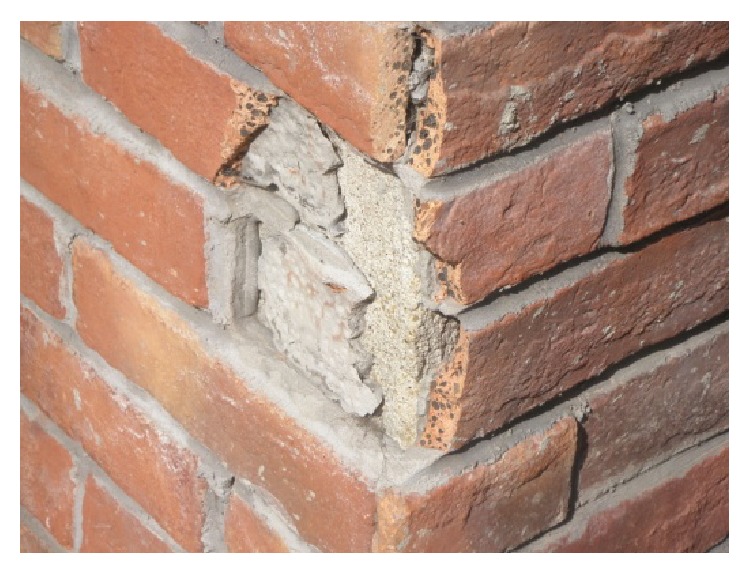
The detail of a wall.

**Figure 10:**
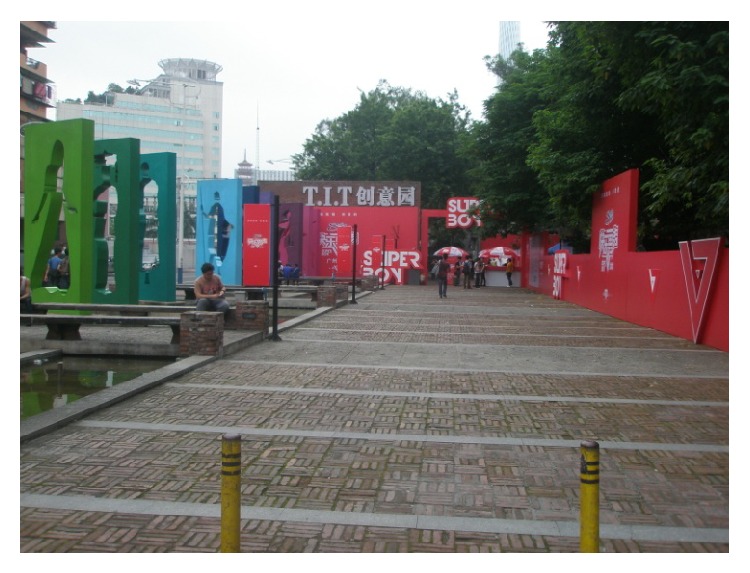
Cultural square of T.I.T Park.

**Figure 11:**
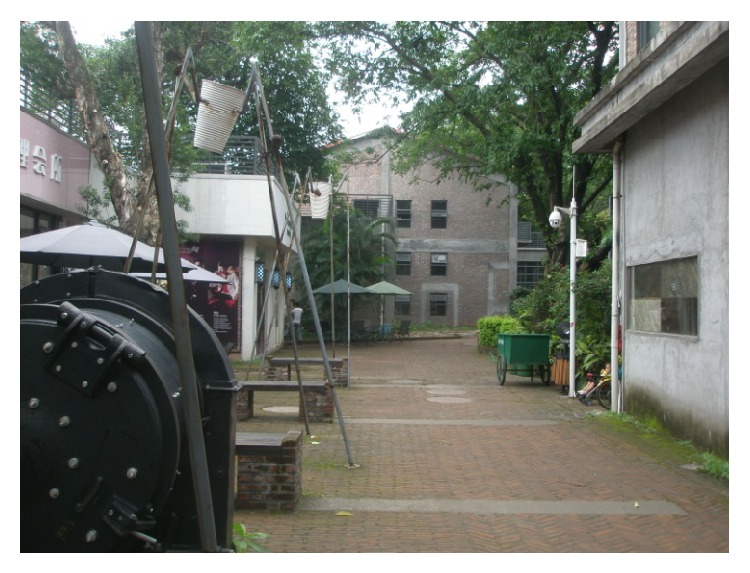
One view of T.I.T Park.

**Figure 12:**
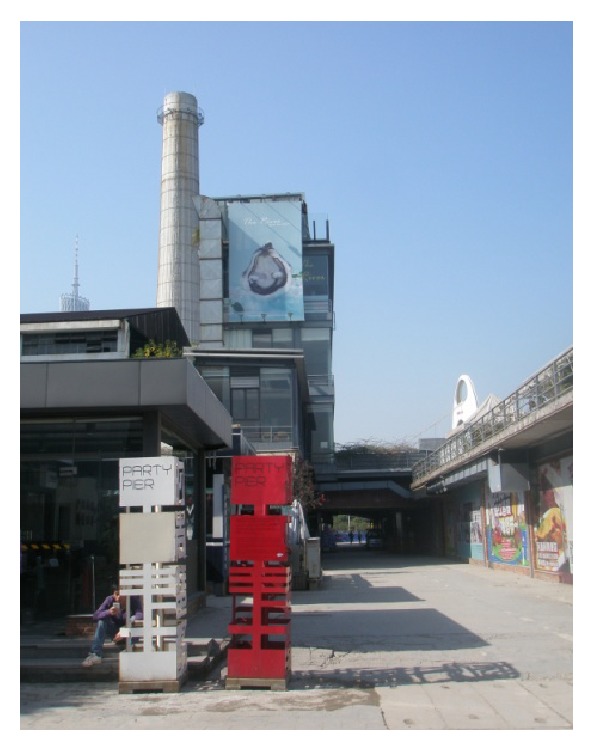
A logo of Party Pier.

**Figure 13:**
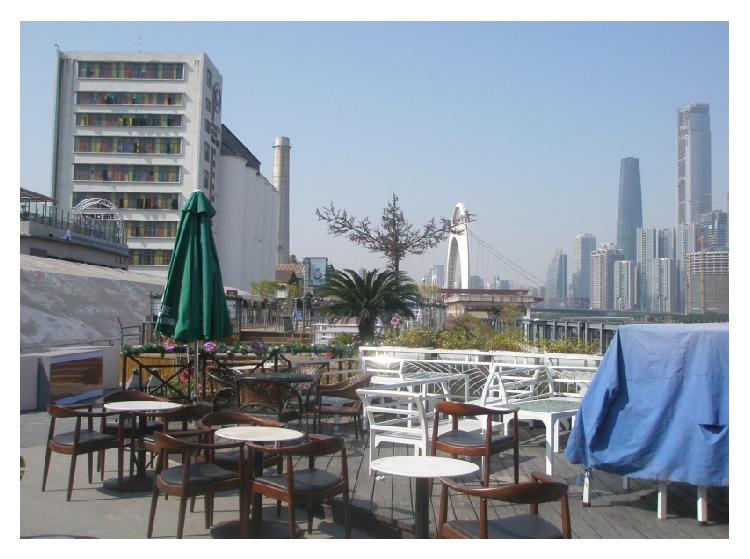
Rriverside view of Party Pier.

**Figure 14:**
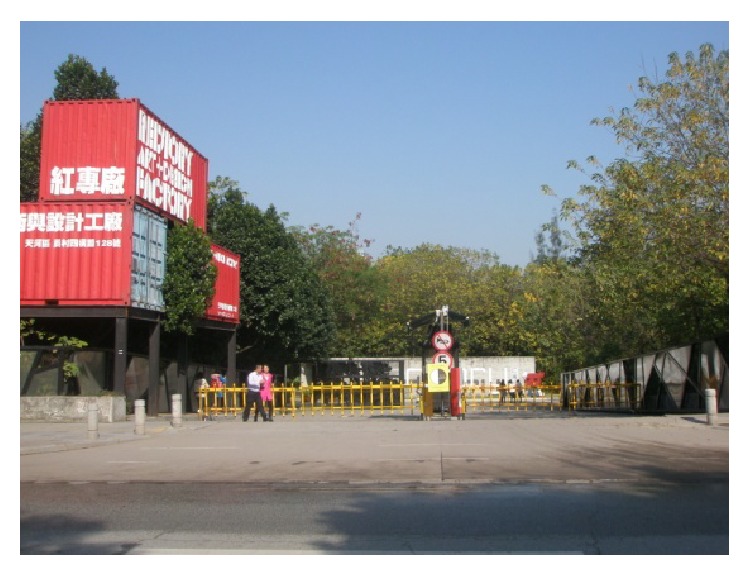
The gate of Redtory.

**Figure 15:**
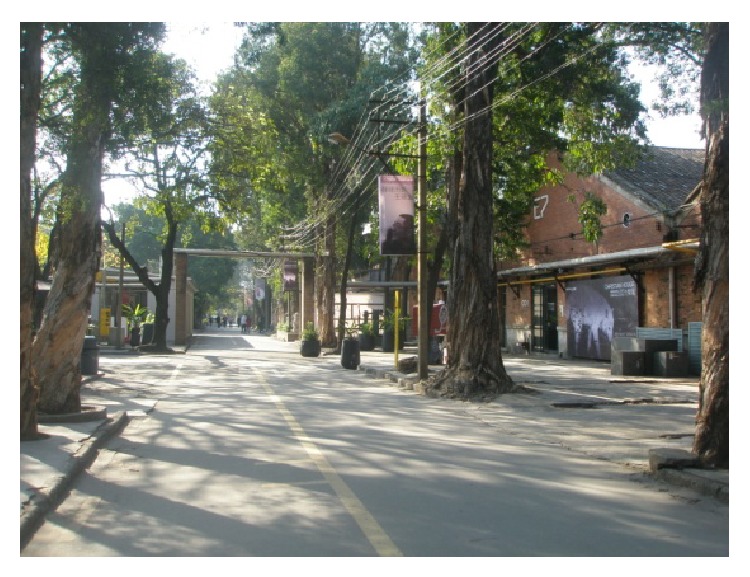
A view of Redtory.

**Figure 16:**
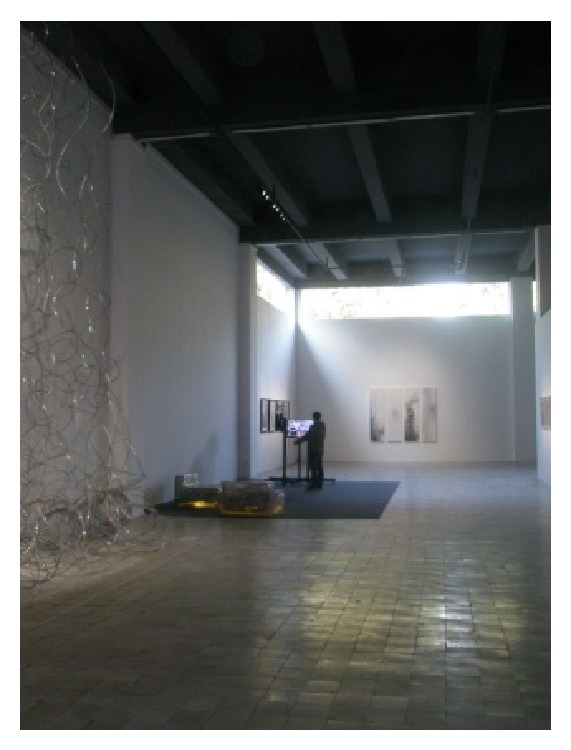
An exhibition hall.

(1) Xinyi International Place which is situated in Fuangcun Avenue East is an early member of “Gold West Bank of Pearl River-Creative Industrial Belt at Riverside” from White Goose Pond to Hedong Bridge. The name “Xinyi” is translated from the meaning of “The German Lutheran Church” which was a cultural landmark in this Guangzhou historic site. After renovation, now Xinyi International Place being with tinges of industrial culture, it extracts elements from old industrial plants with sentimental atmosphere. It integrates modern creative concepts with protecting historic buildings and respecting the original appearance, which achieves the clever fusions of historic buildings and modern decoration style. The place which includes 22 buildings with luxuriant trees combines industrial culture, creative design, photo studio, art gallery, media and advertising corporations, and modern consumption together. The built area of Xinyi International Place is 50,000 square meters and is relatively small, but its panning project is very big and many renovation buildings and new architectures under construction, the completion of which will become a famous cultural brand for Guangzhou, as shown in Figures 2, 3, and 4.

(2) Taikoo Wharf is located in the Guangzhou Zhuhai Gexin Road number 124, in the east side of Pearl River, south of White Goose Pond, north of Hedong Bridge. Taikoo Wharf, also known as Tai Gu Cang, was established by Butterfield & Swire, the predecessor of Swire Group, in the early years of the 20th century. It has witnessed the history of China's foreign trade and served as a wharf and warehouse until 2007. Since then, the wharf's original buildings were preserved and transformed into recreational facilities, including cinema, restaurant, yacht club, gallery, and wine cellar. Taikoo Wharf project covers an area of 71,236 square meters (including land area of 52,500 square meters), wharf coastline 312 meters, total planning construction area 39,599 square meters. The areas of eight warehouses are more than 17,000 square meters, every two warehouses as a group, respectively, divided as four functional areas: wine trade mart, exhibition center, clothing creative design, and retro cinema. By its renewal project, Taikoo Wharf increases recreational walking for nearby residents. On November 13, 2014, Taikoo Wharf held an event named “Guangzhou Night.” The event was an important part of the Guangzhou International Documentary Film Festival and was designed to let the filmmakers know more about Cantonese culture and Guangzhou itself. The theme of this year's Guangzhou Night was the history of Guangzhou as the origin of the Maritime Silk Road (updated: 2014-11-18 10:52, source: http://lifeofguangzhou.com/). As a city's image-promotion campaign, it promotes Guangzhou to the world, as shown in Figures 5 and 6.

(3) Guangzhou Textile Exchange Park (Xingang 82) is located on the Guangzhou Xingang West Road number 82; its predecessors were South China Sewing Machine Factory and Honda Motorcycle Factory. As a Guangzhou demonstration project of “three old renewal projects,” after the transformation, Guangzhou Textile Exchange Park covers an area of 150,000 square meters, with more than 4000 exhibition halls. Its business circle area is the largest as an international textile trade center in Asia. The park has attracted a group of traders, fair business, finance, and facilities, such as mediation, research and development, and quality service, which has basically completed the property rights trading platform, the examination platform, financial services, fashion publishing platform, the creative research and development platform, display exhibition trade platform, and so on. The park managers have forecasted that when the park is completed it will be able to accommodate 1000 enterprises, with 8000 employees, and its turnover wiil be 100 million Yuan or above. In order to do overall design of the park, they brought in a special design team which designed Beijing “798” Art Factory, taking a principle of “repair old as the old,” keeping the original industrial building's facade and combining new elements. Today, when you walk into the park, you will feel a kind of unity and coexistence of history and modernity, as shown in Figures 7, 8, and 9.

(4) Guangzhou T.I.T Creative Park is located in Guangzhou Zhuhai near Kecun subway station. The whole park covers an area of about 93,400 square meters, existing building area 34,300 square meters, its planning building area about 55,000 square meters, and total investment about 200 million yuan, keeping the volume rate less than 0.5 throughout the park, to ensure that the park can give a person a spacious, open, comfortable, and peaceful feeling. Its predecessor is a textile machinery factory site. In a new city's central axis, it is organic integration with the Guangzhou new TV tower. According to the Guangzhou municipal party committee and municipal government proposed industrial adjustment policy and strategy to develop the modern service industry; it sets up a theme of fashion ideas, information collection and release, product display, business communication, fashion leisure, and other functions in a creative industry platform. The park is divided into six big functions such as creative industrial zone, the home of designer, the headquarters and distribution center, brand center, fashion leisure area, supporting service area, and so on. In a process of transforming, the park has been sticking to the principle of “repair old as the old,” respecting history and keeping the textile industry elements. As a classic model of the Guangzhou old factory building reconstruction project in Guangzhou city center, the park is Guangzhou and Zhuhai key construction projects in 2008, gathering many clothing brands, designers, and artists, as a window to show the world with Guangzhou's innovation power, as shown in Figures 10 and 11.

(5) The Zhujiang Party Pier Beer Culture & Art Zone is located on the river bank of Yujiang Road West, Pazhou based on Zhujiang-Inbev Internatioal Beer Museum. It creates a platform for modern beer culture as well as a high-end dining and recreatinal venue with creative architecture above the Modiesha Tunnel and in the riverside area. The freight piers transformed into a transportation pier for Pearl River tour ships and yacht club, creating a place for recreation with ecology and industrial heritage. The zone offers fresh beer all day in term of as main service. Besides the greenway, it is home to Chinese and foreign restaurants, bars, art centers, and creative enterprises. The Party Pier boasts creative venues to hold the corporate activities, commercial performance, wedding ceremonies, cocktail parties, annual meetings, auto shows, press release and conferance, and so forth. The Zhujiang Party Pier as a new hotspot for food and entertainment provides an international city lifestyle, as shown in Figures 12 and 13.

(6) Redtory which is located in Guangzhou Tianhe Yuancun 4 Cross Road number 128 is Guangzhou first nonenterprise, nonproperty, a real meaning of creative area, and an art and life center to international standards. Redtory as international platform with modern view explores new field of art and humanities spirit. Its predecessor was a Guangzhou's food factory which had a glorious history. Its inner space and complex characteristics make it exuding a unique charm. After years of revitalization, it still shows us the glory days of history. Redtory continues to history and also penetrates the creative elements, lets old construction coruscate new vitality and keeps the history of the city texture, lets a person recall and remember the feelings of past era. Redtory sets up a platform for artistic and cultural exchanges, dance studio, international galleries, artist studios, sculpture exhibit hall and square, indoor exhibition hall, auction hall, luxury exhibition hall, private museum, cafe bars, international duty free shop, media organizations, boutiques, health club, flower lane, musical instrument shop, high-end restaurants, internet cafes, import bookstore shops, art and culture experience space, golf clubs, clothing customization, public welfare institutions, and so forth. Redtory sends new creative life culture with beautiful legend, as shown in Figures 14, 15, and 16.

The six sites which are all industrial heritage renovation projects, including the “three old renewal projects” named by Guangzhou municipal government, have some common features. All of them can be called demonstration projects in terms of construction energy efficiency and material recycling. But the technique details among them have some subtle difference, and the aesthetic performances of them are also endowed with different figures. In the next section, based on statistics date, a brief analysis on the issues of construction energy efficiency, old material recycling of six sites, and relevant land uses policy are present. Following that, the paper will further discuss the aesthetic of old material in terms of concepts of “entropy” and “archetype” concerning place making based on mathematic values.

## 3. Analysis on Construction Energy Efficiency, Material Recycling of Six Sites, and Relevant Policy

### 3.1. Material Recycling

The six industrial heritages all remain the old factory buildings or warehouse buildings, totally floor space about 200,000 square meters; the percentages of floor space of these buildings, compared with the 2007 total Guangzhou floor space of constructed building 25,409,200 square meters [17], being about 0.8%. If all this old buildings be demolished, it will create about 240,000 tons of construction wastes; the volume of general industrial solid wastes produced by Guangzhou in 2012 was 614,850 tons [17]; the compared percentages of solid wastes only in these six sites will be about 39%. It will produce great press on environment/ecology in terms of pollution and land waste for landfill. The lifespan of building material was assumed to be 50–70 years, which only refers to the safety of the whole structure; in fact, the service life of most building materials is much longer than that and should be further utilized.

### 3.2. Relevant Policy

The aforementioned six sites all are parts of “three old renewal projects” practices. “Three old renewal projects” proposed by Guangdong provincial government cooperated with the National Ministry of Land and Resources, to promote the economical and intensive land use policy. Guangdong Province is a pilot province for this work and for providing important measures and standards. By 2012, the “three old renewal projects” planned to increase investment by more than 100 billion yuan and more than 2000 hectares of land for these construction purposes. While in 2007, the total construction capital in Guangdong Province was only 101.678 billion yuan (source: Guangzhou Municipal Statistics Bureau, Guangzhou Survey Office of National Bureau of Statistics), so the whole “three old renewal projects” is a big work. For example, Foshan is a Pearl River Delta city nearby Guangzhou; early in 2008, to answer the question how to do with industry transformation, urban transformation and environment reconstruction, and exploring scientific development pattern, Foshan as a vanguard city brought in “three old renewal projects” as guideline, by a flagship project of “Zumiao-Donghuali areas renovation,” also named as Lingnan Tiandi, to offer a Foshan model for “three old renewal projects,” attracting the national attention and praise, and creating stunning appearance of the city. For the renewal projects of “old town, old factory building, and old village in city,” it has confined to building not only external wall decoration and repair, but also the internal management and cultural connotation and to introduce new content for old property.

### 3.3. Technical Lessons and Energy Efficiency

**Figure 17:**
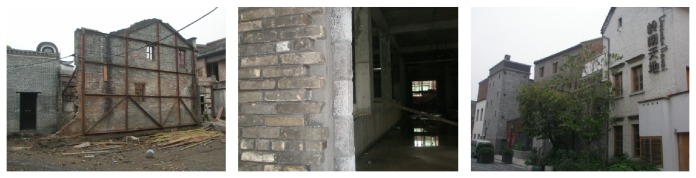
Lingnan Tiandi: conservation and reinforcement; material recycling; culture, taste, and history.

In these cases, the technical details of renovation and material recycling have some lessons to learn. As Figure 17 shows, the Lingnan Tiandi project in order to create an atmosphere of “culture, taste and history,” excepted from conservation and reinforcement of valuable preserving buildings, it still used old materials from old demolished houses in construction of new buildings, obtaining a special aesthetic feelings of historical blocks, to make the entropy of old material itself sending out historical vicissitudes of life. But in Xingang 82, as seen in Figures 8 and 9, the “old wall” is artificial dispose, compared with that of Lingnan Tiandi, not being the original demolished old building materials. The reason is that there are very few old buildings which have been demolished in Xingang 82 renewal projects. If utilizing old materials from other place will increase transportation costs and unlikely suiting the place atmosphere as such as in the Lingnan Tiandi. Transporting distance is a key factor to mitigate the environmental impacts. The material resources that are close to the construction site should be given a priority.

In the product life cycle, the manufacture process emitted the majority of the chemical pollution and consumed most of the energy. Nearly 85% of the chemical emissions were in the manufacture stage. On average, manufacturing process contributes to 90% of energy consumption [18]. The manufacturing stages include raw material extraction, storage, transportation, material fabrication, and waste treatment. While those were not considered because of their minor influence if utilizing old material. Utility of old material also can take another advantage. In terms of old wall enforcement and installation, only simple tools such as a driller and screwdriver are needed to fasten the components of the old material wall, as shown in Figure 17; so the chemical emission and energy consumption of the machines are negligible.

## 4. Explanations of Place Making, Culture, and Aesthetic Performances of Six Sites 

### 4.1. Phenomenology, Place Making, and Culture

The main current concern of material recycling is the decrease of negative impacts such as land waste of landfill and environmental pollution. While from other view, old material utility can create positive effects for place making, especially for historical and cultural atmosphere. Place identity, primarily from the views of environmental psychology, offers a lens through which to ground our understanding of place-based identity and agency in place making. Some literatures confirm that place is not merely a container in which identity is reestablished, embedded, and evolves. Identity is formed and defined in relation to place [19]. The subjective sense of self is defined and expressed not simply by one's relationship to other people, but also by one's relationships to the various physical settings that define and structure day-to-day life. Physical reminders of the past allow one to maintain a sense of continuity with significant places of the past. Continuity with past places is a key to an individual's psychological well-being because of the significant role past places play in the formation and maintenance of self- and group-identity. Research indicates that the personal and group identities associated with and communicated through the local physical environment strengthen the bonds between people and places [20–22].

According to basic views of Husserl's phenomenology, all human conscious states are intentional states, and all intentional states possess essentially correlative intentional objects; that is to say, the subjective consciousness is not essentially something different from objects or phenomena in terms of traditional philosophical views [23], but a correlation of mutual articulation between subject and object. Indeed, just this process constitutes the base for human culture. Undoubtedly the place is a fundamental element for human to correlate this mutual articulation for creating culture.

Culture is not a new word in contemporary era, but in practical aspects in the post-Fordist economy, culture has special meanings. Culture has become an important resource for cities to compete at the regional and international levels. Thus, local elites have used culture as an instrument of urban regeneration and these processes increasingly seek to promote urban branding. Moreover, culture is seen as a way to generate narratives that help cities avoid the perception of standardisation, characterise cities as a unique urban space, and create authenticity, which are necessary elements if a city is to be globally competitive [24]. The joint action of the cultural institutions and representatives of the cultural sector based in the community can turn a place into a brand space with authentic locale feeling that makes a place richer, complex, and tourist-attraction.

Concerning creative city and urban art and culture, we must pay attention to a key word: “place.” A phenomenological theory of planning procedure would encourage planners to note what objects in their communities have meaning and how different frames of reference give different meanings to each object [25]. Phenomenological theories emphasize the “life world,” “direct experience,” and “subjective express,” Phenomenological planning may involve seeing a house not as a merely technological construction, but dwelling; not merely homogeneous and mathematized space, but place; not merely planet raw material, but environment [26]. It concerns offering a unique and characteristic space for neighborhood or community, which produce place identity. In historical sites, the architectural styles of past places of emotional importance have been recreated and/or modified [21] and demonstrated that social, cultural, and physical elements of place support identity and, in turn, engender a strong attachment to place.

### 4.2. Methodology and Approach

The place is a kind of object, but the evaluation of the aesthetic performance of place is subjective process. In phenomenological view, the objective meaning based on subjective intention, that is, the intention, is subjective ability for constituting meaning which refers to phenomenon. The place is a phenomenon condensed life meaning constituted by subjective sense and intention; thus, in order to directly understand the aesthetic performance of place, we make a transition from the objective characteristic of place to the subjective attribution for place making in terms of phenomenology. The aim of this paper is explanation of a common sense about place, not an individual experience, so Jung's key word “archetype” which means collective unconsciousness used in psychology and culture theory is introduced. Bachelard [27] mentioned the relation of a new poetic image to an archetype lying dormant in the unconscious state: this inner immensity that gives their real meaning to certain expressions concerning the visible world. In order to understand the detail of this subjective and phenomenological process, some information-processing theories are also introduced.

According to information-processing theory, the characteristics of cognitive systems include a storage structure acting in following sequence: sensory “store,” short-term memory, and long-term memory. Theorists divide long-term memory into at least three parts: episodic memory, semantic memory, and procedural memory [28]. The episodic memory is a part of long-term memory that stores images of our personal experiences. The semantic memory is that of storing facts and general knowledge. The procedural memory is that of storing information about how to do things. According to the research aim of this paper, the episodic memory is priority for concerns. Because the semantic memory is related with a cognitive framework called schemata that provide networks or categories for connecting ideas and concepts which correlated with archetypes, it is also concerned by this paper, while procedural memory is excluded in terms of its lack of correlation with this research.

First step for analysis on the aesthetic performance of place is transforming place physical characteristics into sensory images based on episodic memory and semantic memory. The sensory images of which we are conscious are not exactly the same as what we saw, heard, or felt; they are what our senses perceived. Perception of stimuli is not as straightforward as reception of stimuli; rather, it involves mental interpretation and is influenced by our mental state, past experience, knowledge, motivations, and many other factors [29]. According to Jung's theories, the mental state, past experience, and so on not only refer to personal state, experience, and so on, but also more importantly and fundamentally refer to collective unconsciousness which is called by Jung as “archetype.” With regard to intention as a key word in terms of Husserl's phenomenology, archetype is a basic factor for intention. Intention is latent ability for constituted meaning, and it is determined by archetype as behind fundamental cause. Unlike attention which is an individual conscious state with direct aim, intention mentioned in this paper is more emphasized as a collective/intersubjective agency with unconscious characteristic without special practical target. So it embody a latent ability to organize senses and provide a framework for understanding that opens up a mental state or senses with recreation, aesthetic, and leisurely atmosphere.

Episodic memory contains images of experiences organized by when and where they happened. The images are important in episodic memory and that cues related to space and time help us to retrieve information from this part of memory. Episodic memories are often difficult to retrieve, because most episodes in our lives are repeated so often that later episodes get mixed up in memory with earlier ones, unless something happens during the episode to make it especially memorable [29]. So if we can maintain episodic memory, which means something “taking place” for us, that is, a place with special characteristic and unique features which must be based on archetype as inner immensity connected past with today and unconscious emotion foundation related to poetic images. The place does not confer the past of his image upon us and yet “by archetype” his peotic image immediately takes root in us.

Archetype is concretization of meanings, making things together and shaping them. Old material is product of general nature process of “entropy.” Because of “entropy” as a universal nature for all things, in terms of phenomenology which integrate subject and object, the “entropy” of material world must relate to the subjective archetype. The archetypical aesthetic meaning of “entropy” on building materials embodies and recalls natural process that reflected a situation for human's being-in-the-world in terms of time's vector. Because of entropy, through the brilliance of a poetic image, the distant past resounds with echoes, and it is hard to know at what depth these echoes will reverberate and die away [27, 30]. The entropy as common process of universe has ontological entity in architectural aesthetics by giving building materials a life beyond some traditional topics concerning material recycling and building sustainable development.

In mathematical calculating of the aesthetic performances of six sites, according to the above-mentioned approach, we use sensory images as subjective attributions replaced the physical/objective characteristics of sites as weighting factors. The factors divide into five sense's parameters, and the values of the weighting are according to the percentages of five sense access to information. Then, we peculiarly pay an attention to the effects of old material in terms of its aesthetic performance for making some special places.

### 4.3. Aesthetic Performances of Six Sites

By the aforementioned reason, especially in emphasizing historical and cultural feelings, the entropy of old material plays a big role. The aesthetic performance of place has much to do with old materials.  Table 3 gives the aesthetic performance values of six places based on weighted different sense. The percentage of human's five senses access to information is individual different. In general, according to the data analyzed by Harvard Business School, researchers show that it is about taste 1%, touch 1.5%, smell 3.5%, hearing 11%, and vision: 83%. We use it as the weighted for calculating the aesthetic performance of the five senses:

(1) P=∑xw,

(2) Pv=∑vf.

In (1), “*x*” is the performance value of different senses; “*w*” is their weighted value. In (2), *P*_*v*_ is aesthetic performance of vision; it includes five weighted factors (“*f*” is a weighted value): *P*_*v*1_ = spatial shape (35%), *P*_*v*2_ = symbol (30%), *P*_*v*3_ = color (15%), *P*_*v*4_ = fabric (10%), and *P*_*v*5_ = environment (10%), where *P*_*v*1_ (spatial shape) can divide as spatial identification, spatial enrichment, scale, and proportion as such. Given exact figures have not accurate and special meaning, the *P*_*v*1_ is a general estimated figure based on survey and interview, and following figures are as similar as this. The *P*_*v*2_ (symbol) refers to the detailed decoration recalled old acquaintances, the effects of which very much related with the entropy of materials. The *P*_*v*3_ (color) is also much affected by the entropy of materials in terms of its influence on color harmony and fulfilling and sentimental atmosphere. The *P*_*v*4_ (fabric) is directly determined by the entropy of materials in terms of its added effects on produce of nostalgia and place attachment. The *P*_*v*5_ (environment) refers to site's nearby landscape. All values of different senses's aesthetic performanc value from 0 to 1. The value below 0.6 is underqualified, from 0.6 to 0.7 is qualified, from 0.7 to 0.8 is overqualified, from 0.8 to 0.9 is good, and above 0.9 is excellent. From the above-mentioned, the vision is the most important senses for place making, audition have some influence, while other senses are nearly negligible in terms of positive effects for place making and poetic image creation. But we must pay an attention to the fact that the smell, touch, and taste can impose very strong negative impact on a place for indicating a place disorder and not suitable for living. The old materials have very strong influence on vision's compositional parameters such as *P*_*v*2_ (symbol), *P*_*v*3_ (color), and *P*_*v*4_ (fabric), as well as *P*_*v*5_ (environment), but with exception of *P*_*v*1_ (spatial shape). The old material has some influence on audition; the hearing in old material environment should have more soften and harmony sounds than new material environment. The old material can mitigate the negative impacts of smell, touch and taste more efficient than new material because of less chemical emission if the buildings be properly repaired and renovated. So the total percentages of aesthetic performance's influence scopes concerning old material are about 60%; it is a deterministic element for place making with historical and cultural atmosphere.

### 4.4. Limitations

One of the limitations of this study is the lack of accurate measure for calculating the aesthetic performance value of material entropy. So the values in Table 3 are based much more on subjective convey and only have some reference significance, not precisely objective values because it faces a difficulty to propose a solution for interdisciplinary problems. Further, some projects are not completely finished or extension projects are still under construction, which add another correlate difficulty. Therefore, all these values and parameters should be explored in future comprehensive studies. Another limitation may be about place and culture. Researchers, cultural creators, and publics now more prominently recognize place, as a crucible for cultural expression. Under the rubrics of creative cities and creative place making, artists and arts and cultural leaders must walk out of their doors, partnering with others, and bring their talents to bear on community [31]. Markusen [31] structures these issues around some overarching research questions that challenge us to move this agenda forward. (1) What are the urban missions of arts and cultures? (2) How do arts participants/consumers behave with respect to place, not just in patronage patterns but also in decisions on where to live? (3) How do arts creators (artists and designer) and arts-provisioning organizations (private and nonprofit) decide where to locate? (4) How individual and organizational actors make a choice in response to a complex and dynamic urban environment? Many lay people as well as academics believe that a key mission of arts and culture is gentrification. In fundamentally, the culture of city must ensure that citizens are well-served in their “right to an expressive life.” But in Lingnan Tiandi project, the process of gentrification led indigenous resident displacement and disbelonging, being a marginalized communities. So the “authentic” culture became a problem that cannot yet be fully explored in this paper.

## 5. Conclusions 

The paper is based partly on field research and systematic observations in each site: photographing and mapping the configuration of business as in each site for an objective record of physical arrangement, recording the conduct of people on these places, and noting interactions between the workers and visitors.

The combined technical activities with cultural/aesthetic values and cooperation with different stakeholders could nurture new thinking and more green innovation. The green building technology become a common view in today low-carbon society and be taken by government as a policy, but the place making and culture are more abstract issues: we still have a limited understanding of the mechanisms behind the aesthetic feelings about a place, that problem should not be left unanswered. In addition, in many regions in china, there are still not enter the postindustrial societies, the people in those regions are not so much “cultural man” as in the developed regions. They not so much realize the value of historical buildings and old materials, and many old buildings have been demolished and treated as wastes. So this paper response to these specific questions also offers both the opportunity and the incentive to address two general sets of issues, relating to the past and future impacts of material recycling and place making.

Why the historic sites have such beauty, mostly because of the entropy of material by which architecture resonance with heaven and earth that constituted an architectural archetype. The entropy of material is produced by nature and cannot be man-made, so the old material is unique and precious resource for some special architecture. The six industrial heritage's renovations included in Guangzhou's “three old renewal projects” realized this view and took this advantage. In these industrial heritage sites, redevelopment of such grey-field sites into new social spaces does not mean that it constitutes gentrification, at least as it is traditionally defined. Most of the articles concerning gentrification describe the plight of the indigenous residents and justifiably asks for a more just means of managing the change in the structure of cities [32, 33]; gentrification signifies a new and distinct dimension of urban sociospatial structure and “gentrification as evidence of vast urban restructuring” [34]. Gentrification has been defined as “the production of space for progressively more affluent users” [35] and by implication to the reduction of opportunities for the less affluent. Gentrification is seen to destroy community life for working class families. It is the work of investors commodifying neighbourhood attributes and displacing the poor [36]. The process involves “invasion-succession” displacement with evictions. That is the story similarly happened in the Lingnan Tiandi in Foshan, but not in above-mentioned six industrial heritage's renovations in Guangzhou, fortunately because there is simply no indigenous residents. But there are still problems, from Figure 5 we can see, the nearby residential buildings are not harmony with the historic sites at least in terms of landscape. Urban landscape mosaics are often characterized by small land-use patches and high heterogeneity. This disadvantage is gradually recognized by government, planners, and managers. For example, the case of Xinyi Place, specifically the whole “Gold west bank of Pearl River-Creative industrial belt at riverside” as the integral projects demonstrated that: the joint action of the cultural institutions and representatives of the cultural sector based in the community have turned the place into an brand space of “authentic Guangzhou” that will make more local experiential knowledge, emotional significance and tourist-frequented place in combination with historical and cultural narratives.

## Figures and Tables

**Table 1:** The configuration of business in six industrial heritages.

	Predecessors	Main service**s **	Other services	Scale
Xinyi Place	Plant; storehouse	Creative industry; design; photo studio	Catering services; bar; exhibition	Medial
Taikoo Wharf	Wharf; storehouse	Catering services; bar	Yacht club	Small
Xingang 82	Plant	Textile exchange; fashion design	Fashion exhibition; catering services; museum	Big
TIT Creative Park	Plant	Creative industry; design	Catering services	Medial
Party Pier	Plant; pier	Catering services; beer culture	Exhibition; museum	Medial
Redtory	Plant	Creative industry; design; exhibition	Catering services	Medial

Notes: predecessors refer to before renovation.

**Table 2:** The service relation between industrial heritages and the statistic sectors of tertiary industries.

The statistic sectors of tertiary industries	Relation
(1) Wholesale and retail trade	●
(2) Transport, storage, and port	○
(3) Hotel and catering service	●●
(4) Information, transmission, software, and information technology service	○
(5) Financial intermediation	○
(6) Real estate	●
(7) Leasing and business services	●
(8) Scientific research, technical service	●
(9) Management of water conservancy, environment, and public facilities	○
(10) Residents services, repair, and other services	○
(11) Education	●
(12) Health and social work	○
(13) Culture, sports, and entertainment	●●
(14) Public management, social security, and social organizations.	○

Notes: ○ no relation; ● have relation; ●● have strong relation.

**Table 3:** The aesthetic performance values of six places.

	Vision (83%)	Audition (11%)	Smell (3.5%)	Touch (1.5%)	Taste (1%)	Total (100%)
Xinyi Place	0.747	0.105	0.032	0.015	0.008	0.907
Taigu Wharf	0.498	0.099	0.028	0.014	0.008	0.647
Xingang 82	0.664	0.066	0.021	0.009	0.006	0.766
TIT Creative Park	0.706	0.097	0.031	0.014	0.007	0.855
Party Pier	0.739	0.077	0.020	0.015	0.009	0.860
Redtory	0.722	0.108	0.032	0.012	0.008	0.882
